# Deletion of the cruciform binding domain in CBP/14-3-3 displays reduced origin binding and initiation of DNA replication in budding yeast

**DOI:** 10.1186/1471-2199-8-27

**Published:** 2007-04-12

**Authors:** Wafaa Yahyaoui, Mario Callejo, Gerald B Price, Maria Zannis-Hadjopoulos

**Affiliations:** 1 McGill Cancer Centre, 3655 Promenade Sir William Osler, Montreal, Quebec H3G 1Y6, Canada; 2 In memoriam

## Abstract

**Background:**

Initiation of eukaryotic DNA replication involves many protein-protein and protein-DNA interactions. We have previously shown that 14-3-3 proteins bind cruciform DNA and associate with mammalian and yeast replication origins in a cell cycle dependent manner.

**Results:**

By expressing the human 14-3-3ε, as the sole member of 14-3-3 proteins family in *Saccharomyces cerevisiae*, we show that 14-3-3ε complements the *S. cerevisiae Bmh1/Bmh2 *double knockout, conserves its cruciform binding activity, and associates in vivo with the yeast replication origins ARS307. Deletion of the α5-helix, the potential cruciform binding domain of 14-3-3, decreased the cruciform binding activity of the protein as well as its association with the yeast replication origins ARS307 and ARS1. Furthermore, the mutant cells had a reduced ability to stably maintain plasmids bearing one or multiple origins.

**Conclusion:**

14-3-3, a cruciform DNA binding protein, associates with yeast origins of replication and functions as an initiator of DNA replication, presumably through binding to cruciform DNA forming at yeast replicators.

## Background

Several studies have shown that cruciform DNA forms at or near eukaryotic origins of replication and is involved in the regulation of initiation of DNA replication [1,2]. In mammalian cells, cruciform extrusion is cell cycle regulated and reaches a maximum at the G_1_/S boundary [3]. The formation of cruciform DNA has also been shown in the *Saccharomyces cerevisiae *replication origin ARS307 [4], as revealed by specific anti-cruciform DNA monoclonal antibodies (mAbs) [5,6]. These mAbs have an enhancing effect on DNA replication, presumably through stabilization of cruciform structures forming at replication origins, which may function as recognition sites for the binding of regulatory proteins of DNA replication [1].

We previously purified from human (HeLa) cells a cruciform-specific binding protein, CBP [7] which was identified by sequence analysis as a member of the highly conserved 14-3-3 protein family [8]. The 14-3-3 proteins are mono- and hetero-dimeric molecules, conserved through plants, invertebrates, and higher eukaryotes (reviewed in [9]). There are at least seven distinct 14-3-3 genes in vertebrates giving rise to nine isotypes (α, β, γ, δ, ε, ζ, η, σ, and τ, with α and δ being phosphorylated forms of β and ζ, respectively), while at least another twenty have been identified in yeast, plants, amphibians and invertebrates (reviewed in [10]). The 14-3-3 proteins are implicated in multiple cellular processes including signalling, cell cycle control, apoptosis, transcription and DNA replication (reviewed in [10-12]).

In mammalian cells, CBP activity occurs only when 14-3-3 is in a dimeric form [8]. Hydroxyl radical footprinting analysis localized the interaction of CBP with cruciform DNA to the four-way junction [13], conforming to the crystal structure of 14-3-3 [14,15], whereby the 14-3-3 dimers form a groove which provides the cruciform binding domain [13]. A similar interaction with the cruciform DNA has been found for the anti-cruciform monoclonal antibody 2D3 [16], suggesting the involvement of a common specific domain in both the 2D3 antibody and the 14-3-3 protein. Alignment of 2D3 antibody and 14-3-3ε sequences revealed two domains of homology within the N-terminal residues of 14-3-3 protein, with 40% similarity for positive residues in the α5-helix (data not shown). This domain is conserved in all 14-3-3 homologues and is located in the amphipathic groove, which is involved in nearly all the interactions of 14-3-3 with its protein partners [17], as well as in the binding to cruciform DNA [13].

In addition to its cruciform binding activity, CBP/14-3-3 was also shown to bind to mammalian replication origins in a cell cycle dependent manner, reaching a maximum at the G1/S boundary [18,19]. The role of 14-3-3 in DNA replication, through direct association with replication origins, has not been determined yet. Proteomic analysis, however, indicated that 14-3-3 interacts with replication initiator proteins such as MCM3, Ku antigen [20,21] and MCM10 [22], implicating 14-3-3 in the regulation of initiation of DNA replication. The function of 14-3-3 proteins as a major regulator of the cell cycle has been reported in many studies. Thus, 14-3-3s regulate the cell cycle progression by sequestrating cell cycle proteins, like CDC25C [23] and p27 [24,25] in the cytoplasm. It has been also shown that 14-3-3 proteins have a temporal role in G1 arrest and S-phase checkpoint function [26].

The involvement of 14-3-3 in DNA replication has also been demonstrated in lower eukaryotes. In *Saccharomyces cerevisiae*, Bmh1 and Bmh2, the budding yeast 14-3-3 homologues [27,28], also bind to cruciform DNA and associate *in vivo *with origins of replication [4]. In a recent study, 14-3-3 was also shown to be regulating the G1/S transition in the budding yeast (*S. cerevisiae*), thus implicating it in the initiation of DNA replication [29]. The nature of replication origins (replicators) and the proteins that interact with them are key to understanding the mechanism regulating this process, which is conserved among lower and higher eukaryotes [30]. The replication origins of budding yeast cells have been well-characterized and used extensively to study the regulation of DNA replication. Furthermore, the genetic manipulations afforded by the yeast cells have made them very useful as host organisms to investigate the function of various proteins.

Taking advantage of these properties, here we analyzed the function of the human 14-3-3ε protein, as the sole member of 14-3-3 in the *S. cerevisiae *cells, in terms of its cruciform binding activity and involvement in the initiation of DNA replication. The human 14-3-3ε protein has more than 60% sequence similarity at the amino acid level with the budding yeast 14-3-3 protein homologues, Bmh1 and Bmh2. Deletion the α5-helix in 14-3-3ε, as a potential cruciform binding domain, resulted in a markedly reduced yeast cell growth, as well as a decrease in cruciform binding activity and in the association of 14-3-3 with the yeast replication origins ARS307 and ARS1. Furthermore, the mutant cells showed a reduced ability to stably maintain plasmids bearing one or two origins. These results suggest that 14-3-3 is regulating DNA replication at the initiation level.

## Results

### Expression of the wild-type and α5-deleted human 14-3-3ε proteins in *bmh1-bmh2 *double knockout yeast cells

The *S. cerevisiae *strain (GG1259), containing a genetic deletion of both the Bmh1 and Bmh2 proteins, is not viable. The viability of this strain is supported by the 14-3-3 *Arabidopsis thaliana *homologue protein, GF14, under the control of the inducible GAL1 promoter [31]. The wild-type and mutant (α5-helix deleted) human 14-3-3ε cDNAs were introduced in GG1259 cells, carried by the pEVP11-URA vector. This vector contains the constitutive ADH promoter, whose expression is increased in media containing glucose as a carbon source. The genomic uracil cassette in the GG1259 strain was removed to allow selection of pEVP11-URA^+ ^clones. The new strains expressing the wild-type 14-3-3ε (14-3-3ε) or α5-helix-deleted 14-3-3ε (14-3-3-α5) respectively, as the sole members of 14-3-3 proteins, were obtained by shuffling the plasmid carrying the *GF14 *gene.

**Figure 1:**
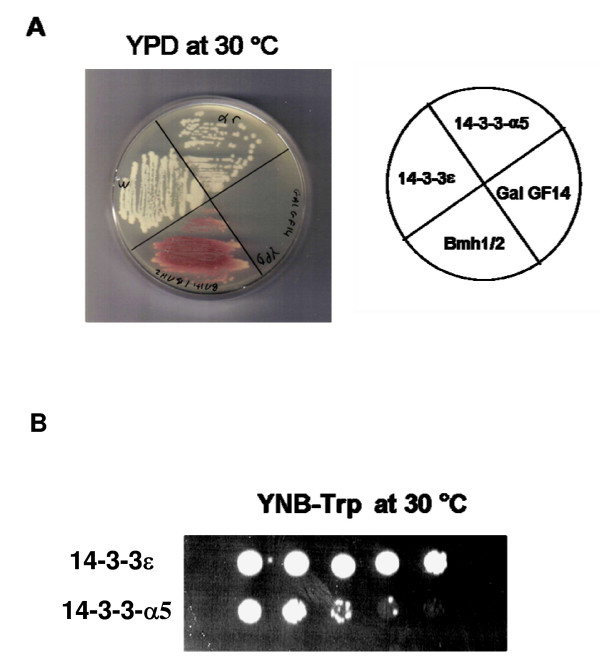
**Growth profile of wild-type and mutant 14-3-3ε proteins**. **A**- The strains expressing wild type 14-3-3ε protein (14-3-3ε), mutant 14-3-3ε protein (14-3-3-α5), GF14 protein (GalGF14) and BY427 expressing Bmh1 and Bmh2 proteins strain (Bmh1/2) (used as a positive control), were streaked on a YPD plate and incubated at 30°C for 3 days. **B**- Cells were grown in YNB media, spotted onto YNB-Trp plates in 10-fold serial dilutions, and incubated at 30°C.

As shown in Figure 1A, both the wild type and mutant strains, heretofore named 14-3-3ε and 14-3-3-α5 respectively, were viable, indicating that both the wild-type and the α5-helix-deleted 14-3-3ε complemented the *S. cerevisiae bmh1-bmh2 *double knockout. This strain (GG1259) can be complemented by several plant and human 14-3-3 homologues, some of which complement well and some very poorly [32]. The cells with GF14 alone (GG1259), named Gal GF14, on the other hand, were unable to grow in YPD media (Fig. 1A), ruling out the possibility of the participation of GF14 protein in the cell growth. As a positive control, Bmh1-Bmh2 wild type cells, named Bmh1/2, were used to verify the yeast cell growth in normal conditions (Fig. 1A). To determine the effect of the mutation on cell cycle progression, both the wild type and mutant strains were grown in YPD and CSM-Trp (synthetic complete medium lacking tryptophan) media at 30°C. In YPD, the mutant grew more slowly than the wild type strain. This slower growth was more pronounced in the selective (CSM-Trp) media (data not shown). The deletion of α5-helix in 14-3-3ε conferred a slow-growth phenotype to the mutant cells compared to wild type, as seen when the cells were grown in YNB media, spotted onto YNB-Trp plates in 10-fold serial dilutions, and incubated at 30°C (Fig. 1B).

**Figure 2:**
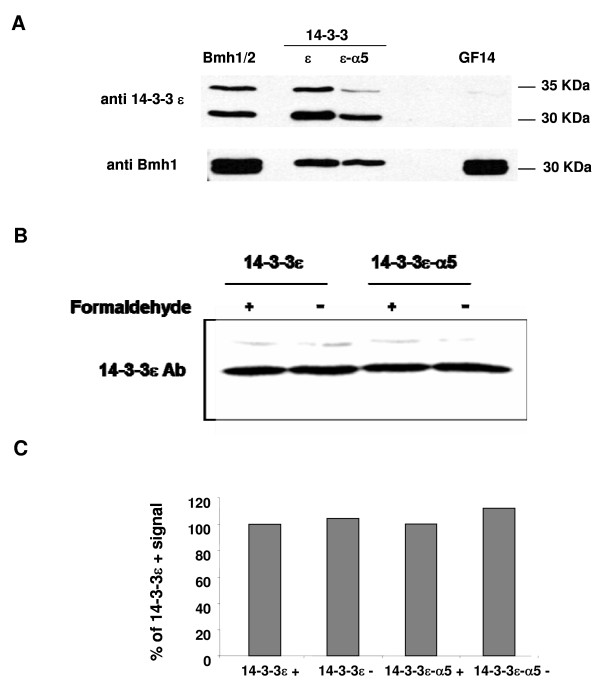
**Western blot analysis of wild-type and mutant 14-3-3ε proteins**. **A**- Whole cell extracts (WCE) of the wild-type(14-3-3ε), the α5-helix-deleted (14-3-3-α5) 14-3-3ε, as well as Bmh1/Bmh2 and GF14 proteins-expressing cells, were prepared and 20 μg of total protein of each were subjected to electrophoresis on a 12% SDS polyacrylamide gel. All proteins were revealed by probing with anti-14-3-3ε antibody and anti-Bmh1 antibody. **B**- Expression of the wild type and the mutant 14-3-3ε in the presence and absence of formaldehyde, detected by anti-14-3-3ε antibody. **C**- Quantification of the immunoblots after normalization for the formaldehyde crosslinked 14-3-3ε.

Having established that the viability of the yeast cells can be fully or partially supported by the wild type or the mutant (α5-deleted) 14-3-3ε respectively, we checked for the presence of these two proteins by western blotting with anti 14-3-3ε antibody (T16; Santa Cruz biotechnology) (Fig. 2A). Surprisingly, both the wild type and mutant 14-3-3ε were detected by this antibody at the position of 30 kDa, indicating that the mutant migrated similarly to the wild type, in spite of the fact that it is lacking 20 amino acids of the α5-helix. A second band of 35 kDa was typically detected by the anti-14-3-3ε antibody, as also previously observed [8,18]. To rule out the possibility that the anti-14-3-3ε antibody recognized a protein other than the recombinant human 14-3-3ε in the yeast background, yeast strains expressing the Bmh1/2 proteins or GF14 protein, were also used as controls (Fig. 2A). Only one of the two yeast 14-3-3 homologues was detected by the anti-human 14-3-3ε antibody, with the same pattern as the human 14-3-3ε, while the GF14 protein was not detected at all. In contrast, when an antibody against Bmh1 protein [4,33] was used in the same blot, both Bmh1 and Bmh2 proteins were detected (Fig. 2A, lower panel, lane Bmh1/2), while only one band was detected in yeast strains expressing the wild type and mutant 14-3-3ε (Fig. 2A, lower panel, lanes ε and ε-α5). This antibody also recognized the GF14 protein (Fig 2A, lower panel, lane GF14), in contrast with the anti human antibody. However, the 2 bands observed in GF14 are present in this protein even when it is blotted with anti-GF14 antibody, as detected in previous studies [4,34]. Thus, these bands are different from Bmh1 and Bmh2, since these 2 proteins are knocked-out in GF14 strain.

We examined next whether the observed growth defect was due to less expression or degradation of the mutant protein. It was recently reported that a cell growth defect in mutant yeast 14-3-3 was associated with a temperature sensitive (ts) phenotype [29]. A similar finding, however, was not observed for the mutant 14-3-3ε in this study (data not shown), which prompted us to verify the amount of 14-3-3ε present in growing (log-phase) cells. Whole cell extracts (WCE) from 10^7 ^cells (OD_600 _of 1.0), were subjected to western blot analysis by immunoblotting with anti-14-3-3ε antibody. The wild-type and mutant proteins were similarly expressed (Fig. 2B,C), suggesting that the mutation did not affect the expression level of this protein, and that any other observed effect in the mutant cells would be specifically functional. Furthermore, since 14-3-3 proteins associate with multiple protein partners as well as with cruciform DNA, we verified whether these associations affected the affinity of the anti14-3-3ε antibody for the protein. WCE from formaldehyde-fixed cells were also subjected to western blotting (Fig. 2B), and quantification of the bands showed (Fig. 2C) that formaldehyde treatment had no effect on the amount of 14-3-3 present in the extracts.

### Effect of α5-helix deletion in 14-3-3ε on cruciform-binding activity

**Figure 3:**
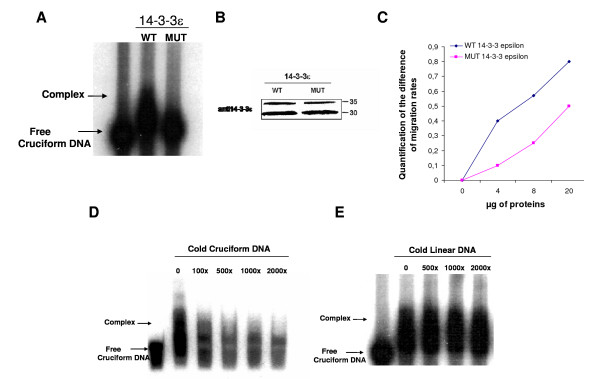
**Cruciform-binding activity of 14-3-3ε**. Band shift assays were carried out as described in Methods. **A**- 3 ng of ^32^P-labelled cruciform DNA was incubated in the presence of 20 μg of wild-type (*lane2*) or mutant (*lane 3*) total whole cell extracts. Free cruciform DNA denotes the position of the probe alone. **B**- Western blot of proteins eluted from band-shifts initially performed with 300 ng of labelled cruciform DNA, and probed with anti-14-3-3ε antibody (see Methods). **C**- Representative protein concentration curve using increasing amounts of total WCE containing wild type or mutant 14-3-3ε proteins incubated with 3 ng of cruciform DNA. Differences of migration rates of the shifted complexes with free cruciform DNA are represented with arbitrary units. **D-E**- Competition binding assays were carried out as described in Methods. Cold cruciform DNA (**D**) and cold linear DNA (**E**) competitors were added in the indicated amounts in a reaction with wild type 14-3-3ε. Free cruciform DNA denotes the position of the probe alone.

Since both the wild-type and the α5-helix-deleted human 14-3-3ε support the viability of the yeast cells, we analyzed whether 14-3-3ε protein, when expressed in yeast, conserved its cruciform binding activity and whether deletion of its potential cruciform domain affected this activity. Comparative band-shift analyses (Fig. 3A), using whole cell extracts from cells expressing either wild-type (lane 2) or mutant (lane 3) 14-3-3ε, indicated that the mutant 14-3-3ε protein had a decreased mobility shift compared to the wild-type. To verify the presence of 14-3-3ε in the band-shifted complexes, the proteins were eluted (see Methods) and analyzed by western blotting, using the anti-human 14-3-3ε antibody. As expected, both the wild-type and mutant 14-3-3 proteins were detected (Fig. 3B), with both the major 30 kDa and the less intense 35 kDa bands being present, as always detected by the anti-14-3-3ε antibody [8,19].

Because the mutation in 14-3-3ε is the sole difference between the two cell extracts, and the fact that 14-3-3 binds physically [8] and specifically [13] to cruciform DNA, the data also suggest that α5-helix is only partially responsible for the cruciform binding activity, since its deletion did not completely abolish it. The reduction in the mobility shift might not be attributable to the shorter length of the deleted protein, as also shown previously in similar studies [35,36]. However, to better characterize the binding of 14-3-3ε to cruciform DNA, we performed a protein concentration curve (Fig 3C). The results show that for both the wild type and the mutant proteins, the shifts are protein concentration dependent and proportional to the amounts of the proteins added. Nevertheless, for any amount of total proteins used, the band shift produced by the mutant strain extract is lower than the wild type, suggesting that the deletion of the α5-helix in 14-3-3ε is responsible for this reduction.

It has been previously shown that CBP/14-3-3 recognizes and binds to cruciform DNA specifically by structure rather than sequence [4,7]. To verify the specificity of the binding to cruciform DNA by the yeast-expressed 14-3-3ε, competitive band-shift assays were performed, using increasing amounts of cold cruciform or cold linear DNA to compete with the labelled cruciform in the binding reaction, as previously described [7]. As shown in Figures 3D and 3E, the mobility shift of the cruciform-14-3-3ε complex decreased progressively with higher molar excess of cold cruciform competitor, and was nearly completely abolished at a molar excess of 500 × (Fig. 3D), while up to 2000 × of cold linear DNA did not compete with the cruciform complex formation (Fig. 3E).

**Figure 4:**
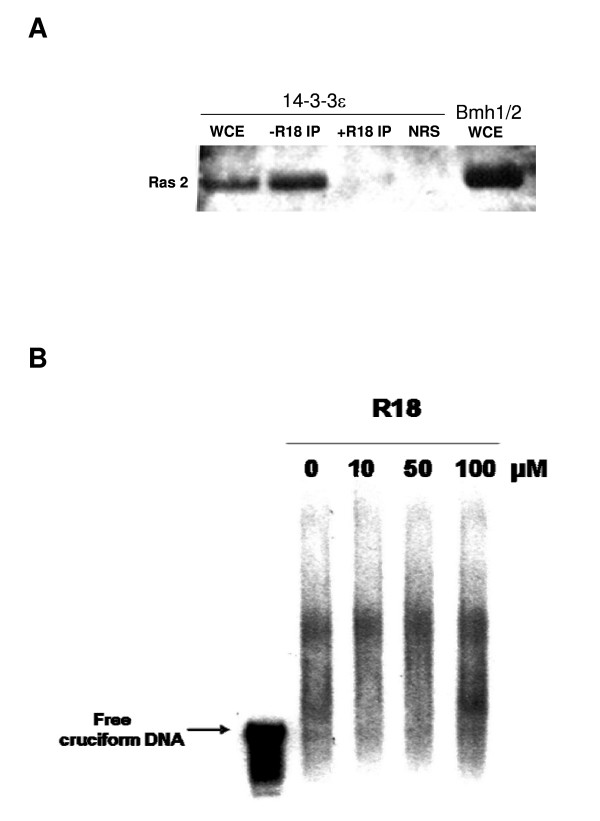
**Effect of R18 on cruciform-binding activity**. Lysate was prepared from yeast cells expressing wild-type 14-3-3ε as described in Methods under the WCE preparation section. **A**- Total protein extract (1 mg) was incubated at 30°C for 30 min in the absence or presence of 100 μM of R18 peptide, followed by immunoprecipitation with anti 14-3-3ε antibody and blotted with anti-Ras2 antibody. Immunoprecipitation with normal rabbit serum (NRS) and a Bmh1/2-expressing strain were used as controls. **B**- 14-3-3ε protein extracts were incubated in the same conditions as in section A with increasing amounts (10, 50 and 100 μM) of R18, and then subjected to a cruciform binding assay, as described previously (see Methods). Free cruciform DNA denotes the position of the probe alone.

Furthermore, to determine whether the inhibition of the cruciform binding activity in the mutant protein might be due to a disruption of the site involved in the interactions of 14-3-3 with its protein partners, which is located in the groove [17], we analyzed the effect of addition of the 14-3-3 peptide inhibitor, R18 (Biomol International, LP) on the 14-3-3ε expressing yeast extract. In mammals, R18 binds with the same affinity to all 14-3-3 isoforms and disrupts their substrate binding [37]. A concentration of 25 μM of R18 has been shown to inhibit the interaction of 14-3-3 proteins with their proteins partners in HeLa cells extract [38]. However, since there is no previous characterization of the effect of R18 in yeast extract, we analysed the ability of this inhibitor to disrupt the interaction of 14-3-3ε with Ras2 protein, a known 14-3-3ε binding partner [39]. The results show clearly that the yeast Ras2 protein immunoprecipitates with 14-3-3ε in the absence of R18, as indicated in the western blot by the band at 40 kDa, while this band is completely absent after addition of 100 μM of R18 (Fig. 4A). WCE from a Bmh1/2 expressing cells was used as a positive control. These data suggest that R18 functions in yeast as an inhibitor of 14-3-3 interactions, in the same way as in mammalian cells.

Based on these results, we next analyzed the binding activity of the wild-type 14-3-3ε in the presence and absence of R18. Even after using an excess amount (up to 100 μM) of R18 in the cruciform binding assay, band shift analyses showed that the cruciform binding activity of 14-3-3ε is not affected by R18 treatment (Fig. 4B). Addition of very high concentration of R18 (100 μM) resulted in higher intensity of the same complex seen in the absence of R18 (Fig. 4B, last lane). These data suggest that 14-3-3ε binds to cruciform DNA even when its protein binding site is inhibited by R18 peptide, ruling out the possibility that another 14-3-3 partner is involved in this activity. Thus, overall the data suggest that the reduced cruciform binding activity of the mutant 14-3-3ε is specifically related to the deletion of the α5-helix.

### Association of wild-type and α5-helix deleted 14-3-3ε with yeast replication origins

**Figure 5:**
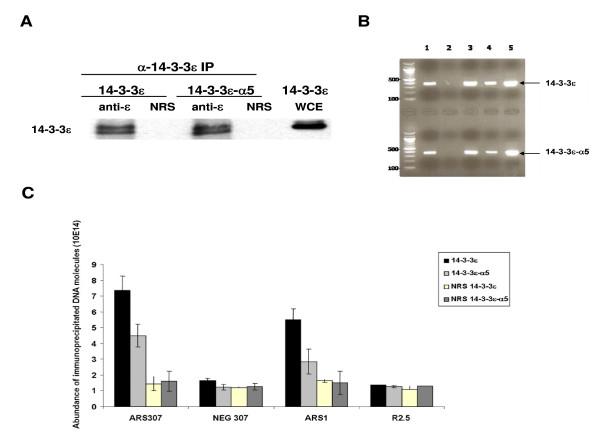
**Chromatin immunoprecipitation assays**. **A**-Formaldehyde cross-linked WCEs were immunoprecipitated with anti-14-3-3ε antibody. Approximately the same amount of wild type and mutant 14-3-3ε were revealed by the anti-14-3-3ε antibody. NRS and 14-3-3ε WCE were used as controls for the IP. **B**- The immunoprecipitated chromatins were subjected to conventional PCR and resolved in a 1.5% agarose gel. Upper panel of the gel shows the PCR products of the ChIP performed with wild-type 14-3-3ε extracts (arrow). The lower panel shows the PCR products of the ChIP performed with α5-helix-deleted 14-3-3ε extracts (arrow). Template DNAs from reverse cross-linked cells immunoprecipitated with anti-14-3-3ε antibody (lane 1) or pre-immune sera (lane 2), wild-type and mutant yeast WCE (lane 3 in upper and lower panel respectively); 10 ng of genomic DNA (lane 4) and 10 ng of plasmid pRS306 containing ARS307 (line 5), were used as controls. **C**- The amounts of wild-type or α5-deleted 14-3-3ε proteins associated with ARS307, ARS1 and their negative regions, Neg307 and R2.5 respectively, were measured by Real-time PCR. Cross-linked WCE were immunoprecipitated using anti-14-3-3ε antibody or NRS, and each corresponding ChIP material was used as template DNA. Error bars represent the average of three separate experiments and one standard deviation.

The budding yeast (*S. cerevisiae*) replication origin ARS307 has been well characterized [40,41]. Among other DNA sequence features, it contains an inverted repeat (IR), which extrudes into a cruciform structure, as shown by immunoprecipitation (IP), using the specific anti-cruciform DNA monoclonal antibody, 2D3 [4]. Since both wild type and mutant 14-3-3ε proteins support the viability of the budding yeast strains, we examined their association with the replication origin ARS307 by the chromatin immunoprecipitation (ChIP) assay, using formaldehyde cross-linking and immunoprecipitation with anti-14-3-3ε antibody or normal rabbit serum (NRS), as previously described [4,19]. Extracts from wild type and mutant yeast strains containing the same amount of total protein (1 mg), were subjected to immunoprecipitation using anti-14-3-3ε antibody. The western blot (W.B) shows that in both IPs, the antibody precipitated approximately the same amount of 14-3-3ε (Fig. 5A), while NRS failed to immunoprecipitate it. A WCE containing only 14-3-3ε was used as a positive control (Fig. 5A). These results validated the ChIP assays and have been used as control for the quantification of the immunoprecipitated DNA. The obtained ChIP materials were subjected to conventional PCR using primers specific for ARS307. The results show that for the anti-14-3-3ε antibody ChIP, an expected 370-bp ARS307 fragment was amplified (Fig. 5B, lane 1, upper and bottom panels, respectively). In contrast, PCR amplification of the chromatin that was immunoprecipitated by the pre-immune serum, did not produce this fragment (Fig. 5B, lane 2, upper and bottom panels, respectively), confirming the specificity of the amplification, whereas use of WCE from the 14-3-3ε wild type (lane 3, upper panel) and the mutant (lane 3, bottom panel) strains, or of *S. cerevisiae *genomic DNA (lane 4, upper and bottom panels, respectively) as template DNA did. The plasmid pRS306, containing ARS307 cDNA (lane5, upper and bottom panels, respectively), was used as an additional positive control of the PCR reactions. These data showed that the human 14-3-3ε protein, like its yeast homologues, can associate with the yeast replication origin, ARS307, and indicated the universality of the origin binding activity of 14-3-3 proteins.

To better analyze the effect of the α5-helix deletion in 14-3-3ε on its ability to bind to replication origins, the amount of origin-containing immunoprecipitated chromatin was quantified by Real-time PCR using two different origins (ARS307 and ARS1) and their respective negative regions (Neg307 and R2.5). Cross-linked whole cell lysates, containing either wild-type or α5-helix-deleted 14-3-3ε protein, were normalized to 20 μg/μl and 200 μl were immunoprecipitated with anti-14-3-3ε antibody or NRS. The immunoprecipitated chromatin obtained in each ChIP assay was then quantified by Real-time PCR, using the regions indicated above as template DNA and the specific primers for amplifying each region (see table 1). The quantity of PCR product from each template is calculated at the threshold of amplification determined by the light-cycler software (Roche) by comparison to the values of the genomic standard curve. As shown in Figure 5C, these values (histogram bars) represent the amount of chromatin immunoprecipitated in each sample, reflecting the number of DNA molecules initially used in the Real-time PCR reaction. The results indicated that the association of 14-3-3ε with ARS307 and ARS1 decreased by approximately 40% in the mutant cells, an effect that may be directly attributed to the deletion of the α5-helix in 14-3-3ε, as it is the sole physiological difference between the wild-type and mutant cells. In contrast, in the respective negative regions of ARS307 and ARS1 (Neg307 and R2.5 respectively), the amount of immunoprecipitated DNA was not significantly different between the wild-type and the mutant cells. These amounts were also comparable to the number of molecules brought down non-specifically by NRS in both cell types. Taken together, these data suggest that 14-3-3ε binds specifically to origins of replication in yeast, and that the decreased association with origins in the mutant cells is likely a direct effect of the deletion of the α5-helix in 14-3-3ε.

### Plasmid stability assay

DNA cruciforms have been implicated in the initiation of DNA replication by acting as regulatory signals at origin sites at the onset of S-phase (reviewed in [2]). Furthermore, 14-3-3 proteins have higher cruciform binding (CBP) [2] and origin association [19] activities at the G1/S boundary. In view of these observations, and in order to test the hypothesis that 14-3-3 is a regulatory protein at the initiation level of DNA replication, we analyzed whether deletion of the α5-helix in 14-3-3ε affected the maintenance and initiation of DNA replication of minichromosomes (ARS-containing plasmids). In *S. cerevisiae*, characterization of ARS function was largely done by using a plasmid stability assay [40,41].

Here, the wild type (14-3-3ε) and the mutant (14-3-3-α5) yeast strains were used to measure plasmid loss rates, using a pair of test plasmids, containing either a single ARS, pARS-1 (containing ARSH4) or two, pARS-2 (containing ARSH4 and ARS307) (see Methods). In all the previously characterized mutants of proteins involved in the initiation of DNA replication, such as ORC, Cdc6, MCM, and Cdc45, plasmids containing a single replication origin had higher plasmid loss rates than plasmids containing multiple replication origins, while wild-type cells lost both test plasmids at equally low rates [42,43].

**Figure 6:**
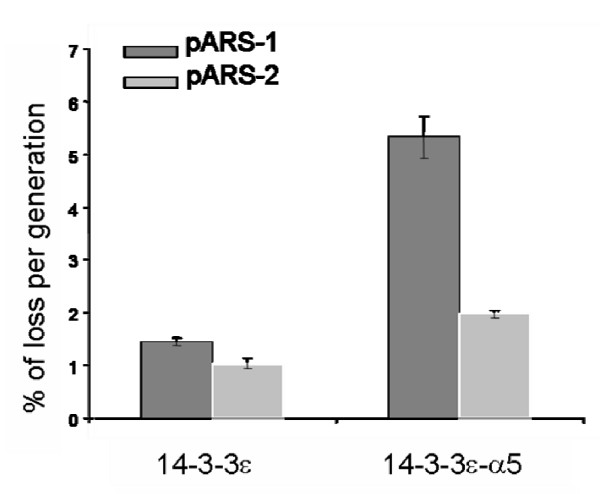
**Plasmid stability assay**. Plasmid loss rates were measured for the wild-type and α5-deleted 14-3-3ε yeast cells containing either pARS-1 or pARS-2. Plasmid loss rates are expressed as the average of percent loss per generation. Error bars represent the average of three separate experiments and one standard deviation.

As shown in Figure 6, deletion of the α5-helix in 14-3-3ε compromised the mutant cells, containing 14-3-3ε-α5 protein, in their maintenance of the minichromosome pARS-1, showing a plasmid loss rate of approximately 5% per generation. In contrast, when pARS-2 was expressed in these cells, the plasmid was more stable by approximately 2.5-fold. When the same assay was performed using wild-type cells, both test plasmids were lost at a much lower rate of approximately 1–1.4% per generation. These results indicated that the deletion of the α5-helix in 14-3-3ε was responsible for the higher plasmid loss rate, suggesting that this protein regulates DNA replication at the initiation level.

## Discussion

In the present study, we analyzed the effect of deleting the α5-helix domain of 14-3-3ε on its cruciform binding and replication origin binding activities as well as on the initiation of DNA replication. The results show that the human 14-3-3ε protein can replace its counterparts, Bmh1 and Bmh2, in *S. cerevisiae*, as yeast cells expressing only human 14-3-3ε were viable. Previous studies have shown that the human cruciform binding protein CBP/14-3-3 associates *in vitro *with cruciform-containing DNA [13] as well as *in vivo *with mammalian origins of DNA replication [19]. The Bmh1 and Bmh2 proteins have been shown to have the same activities as their mammalian homologues, namely the 14-3-3 proteins [4]. CBP association with cruciform DNA occurs only in the dimeric form of the protein [8], mainly in a heterodimeric conformation with a preferential pattern of heterodimerization [44]. Furthermore, using different genetic backgrounds of yeast, it was previously shown that the Bmh1-Bmh2 heterodimer was more efficient in binding to cruciform DNA than the homodimer of either protein [4]. Here, we have shown that the human 14-3-3ε expressed in yeast conserves its cruciform binding activity, indicating that it can dimerize with itself and functions as a homodimer. Previous studies on dimer profile analysis reported that endogenous 14-3-3ε isoform was never found as a homodimer [45,46]. This discrepancy may be explained by the preferential pattern of dimers adopted in mammalian cells, which is not afforded in the yeast strains used in this study. Furthermore, it was previously shown that the 14-3-3 plant homologue GF14 was also able to homodimerize and binds to cruciform DNA [4].

Although the mode of association of CBP/14-3-3 with cruciform DNA has been previously shown [13], the regions of 14-3-3 responsible for this activity have not been determined yet. Thus, hydroxyl radical footprinting analyses showed that CBP binds to cruciform DNA at the four-way junction [13], as does the anti-cruciform antibody, 2D3 [16]. Taken together, these results suggested the involvement of a common domain(s) between CBP/14-3-3 protein and 2D3 antibody responsible for CBP activity. Alignment of 14-3-3ε and 2D3 amino acid sequences revealed a 23% identical (40% identical when positive charges residues are taken in account) region, corresponding to the α5-domain in 14-3-3ε protein (amino acids 119–169). Crystallography of 14-3-3 protein showed that the molecule is a cup-shaped dimer [14,15]. Each monomer contains nine α-helices (denoted α1-α9), with the dimer interface formed from helices α1, α3 and α4, while helices α5-α9 form an amphipathic groove that is considered as the binding domain of the protein [17]. This information argued strongly for a possible involvement of the 14-3-3 α5-domain in cruciform binding activity and regulation of DNA replication, and prompted the analysis of the effects of its deletion on these processes.

As shown above, although both the wild-type and α5-deleted 14-3-3ε were able to bind cruciform DNA, the DNA-protein complex formed with the mutant protein exhibited a lower band-shift than the one formed with the wild-type, indicating an inhibitory effect of the α5-deletion on the cruciform binding (CBP) activity. The α5-domain, however, seemingly participates partially in this activity, since its deletion did not completely abolish CBP activity. Considering that 14-3-3 binds to cruciform DNA in a structure-specific manner [7], the domains adjacent to the α5-helix might contribute to its full activity, as it is the case for the interactions of 14-3-3 with other proteins [17]. Alternatively, the difference in the cruciform mobility-shift might be due to changes in the conformation of the cruciform DNA molecule upon binding to the mutant protein. It was shown previously that CBP/14-3-3 binding can cause structural alterations in the DNA, thereby presenting the appropriate signals for DNA replication [13]. The band-shift patterns obtained in this study may have arisen because the mutated 14-3-3ε might not be able to induce the proper structural alterations in the cruciform forming within the origin, which may either result in a less stable cruciform or alter the ability of other proteins to bind to the origin. Cruciform stabilization at replication origins has been shown to be induced by the anti-cruciform DNA antibody, 2D3 [1].

14-3-3 proteins associate *in vivo *with replication origins in mammalian [18] and yeast [4] cells. In this study, we showed for the first time that a human 14-3-3ε was able to bind specifically to active yeast origins of replication, ARS307 and ARS1, while deletion of the α5-helix in 14-3-3ε decreased its association with these origins. The sequence requirements for replication origin activity vary considerably between different eukaryotic organisms. In *S. cerevisiae*, these sequences consist of an essential A element, containing the ARS consensus sequence (ACS) [47] and two or three important B elements that are collectively required for origin function [40]. Initiation of replication requires the presence of ACS, which together with the B1 element forms the binding site of the origin recognition complex (ORC) [48]. ORC remains stably bound to the chromatin throughout the cell cycle [49], and recruits other proteins to the origin in a cell cycle regulated manner [50]. In higher eukaryotes, the molecular basis of origin identity and function is less well understood. Indeed, the cis-acting sequences required to direct the initiation of DNA replication are more complex and may extend over thousands of base pairs of DNA in mammalian origins [51]. Like in *S. cerevisiae*, these sequences direct the formation of a number of protein complexes leading to the assembly of the pre-replicative complex (pre-RC) at origins during G1 [30]. These proteins include, among others that are conserved, ORC homologues that have been identified in all eukaryotes [52]. The sequence preference for ORC binding is not absolute in metazoa, suggesting that there may be other factors that increase the affinity of ORC binding to origins [53]. These factors include proteins such as the recently identified initiator protein OBA/Ku86 [54]. Although there is no apparent sequence homology between mammalian and yeast origins, previous studies as well as the data here suggest that 14-3-3 binds to a common structural feature in these different origins, namely cruciform DNA. Furthermore, considering that 14-3-3 is a cruciform binding protein, and the fact that several isoforms of this protein have been immunoprecipitated with cruciform structures present in mammalian and yeast replicators [4], it may be concluded that 14-3-3 proteins bind to active yeast and mammalian origins at cruciform structures.

In this study, we have also identified 14-3-3 as a novel initiator protein for yeast DNA replication. 14-3-3 proteins are known to be important regulators of vital cellular functions such as transcription, cell cycle control and signal transduction. It is thus reasonable to assume that the replication initiation phenotypes of the α5-helix-deleted 14-3-3ε might be the indirect result of an aberrant transcription or regulation of other initiator proteins. The role of 14-3-3 in the regulation of transcription in *S. cerevisiae *has been recently investigated, using a bmh2-ts mutant [33]. That study revealed that the yeast 14-3-3 homologues stimulate the transcription of many genes involved in ergosterol metabolism and in stress response, but found no alteration in the transcription of initiator proteins like ORC, Cdc6 or MCM proteins. 14-3-3 has also been shown to regulate the transcription of inducible genes by regulating the function of the histone H3 protein [55]. However, the association of 14-3-3 with DNA bound to histone H3 cannot explain the decrease of the association of the mutant 14-3-3 with the origin in this study, because the region of the chromosome that is affected is limited to origin containing DNA. Furthermore, the association between 14-3-3 and histone proteins is not systematic. In a recent study, a GINS complex responsible for fork progression during DNA synthesis was purified [56] and found to contain the four histone proteins but no 14-3-3. In general, the notion that 14-3-3 may play an indirect role in replication by controlling transcription or even protein-protein interactions would be incompatible with the fact that 14-3-3 physically interacts with replication origins, as shown previously [19] and here by the ChIP assay. Furthermore, the data in this study indicate that 14-3-3ε plays a direct role in the initiation of DNA replication, as it is required for the maintenance of a minichromosome, as assayed by plasmid loss rate of two ARS-containing plasmids. This assay can reveal whether a mutant is likely defective in the initiation of DNA replication [42].

Lottersberger and co-workers [29] showed in a recent study that some bmh1-ts mutants had growth defects, which may be related to a low expression of G1 cyclin-cdk1. They attributed the cell cycle arrest to a decrease in the level of CLN2 protein (one of Cdk1 subunits) in the bmh1 mutants, where there was a complete G1/S arrest after shifting to 37°C. This statement was not necessarily true for the other mutants used in the same study, where the G1/S transition was only delayed. In our study, we observed a slow growth in the 14-3-3ε-α5 protein expressing cells. This growth defect is most likely not attributable to a lower expression of the G1 cyclin-cdk1, since our mutant cells are not temperature sensitive and do not represent any noticeable difference in the cell cycle distribution in log phase (data not shown). Rather, it might be explained, at least in part, by the results from the plasmid stability assays, given the extent of the loss viability observed in the mutant cells. This assay indicates that the mutation in 14-3-3ε is affecting the initiation of DNA replication most likely by disrupting its direct association with origins of replications sites. Nevertheless, given the effect of 14-3-3 proteins on different regulatory processes, such as transcription and cell cycle progression, it is possible that other mechanisms also contribute to this growth defect.

## Conclusion

Overall, the data presented here attest to the universality of binding of 14-3-3 proteins to replication origins and their involvement in the regulation of DNA replication. Our data, coupled with the apparent function of 14-3-3 proteins in the G1/S transition [19], and the role of cruciform DNA in the initiation of replication [1], suggest that 14-3-3 may function in the initiation of DNA replication by regulating the formation or maintenance of the pre-RC through interactions with cruciform DNA. To elucidate the exact mechanism of this process, the association of 14-3-3 with other initiator proteins, as well as the cell cycle profile and regulation of these proteins in budding yeast are currently being investigated.

## Methods

### Strains, plasmids and constructs

The yeast strain *GG1259 *(*haploid leu2-3,112 ura3-52 trp1-92 bmh1*::*LEU2 bmh2*::*URA3 his4 *pYES-TRP(GF14); a generous gift from Dr. G. P. H. van Heusden, Leiden University, The Netherlands) was used. The strain was modified by disrupting the URA cassette and selected by 5-fluoroorotic acid, 5-FoA (Wako Pure Chemicals, Osaka, Japan), and used as a host for the transformation by pEVP11 plasmid [57] bearing either wild-type or mutant 14-3-3ε cDNAs. pEVP11 contains the ADH promoter and has uracil as a selection maker. pYES(*GF14*) plasmid, initially present to rescue the *bmh1-bmh2 *double knockout, was shuffled from the cells. The resulting new strains expressing 14-3-3ε or 14-3-3ε-α5 proteins were used as genetic backgrounds for the rest of the experiments. YPD and complete minimal dropout medium with a further addition of the specific nutrient requirement were used when mentioned. All cell cultures were grown at 30°C.

### Test plasmids-pARS-1 and pARS-2

The episomal plasmid pRS313-HIS3 contains ARSH4 as a yeast origin of replication. It has been used without any modifications as test plasmid pARS-1, or modified by the insertion of ARS307 sequence to construct the test plasmid pARS-2. pARS-2 was constructed by cloning, in the pRS313 multiple cloning site, a 365 bp EcoRI-SalI ARS307 fragment which was initially amplified from the pRS306 plasmid bearing the ARS307 sequence (a gift from Dr. C S Newlon, Univ. of New Jersey Medical School). The PCR amplification of this fragment was done by using the PARS307 primers (Tab. 1). Successful cloning of the ARS307 fragment in pARS-2 was verified by sequencing.

### Deletion of the α5 domain in 14-3-3 epsilon protein

Full-length human 14-3-3ε cDNA (a gift from Dr. A. Aitken, Univ. of Edinburgh) was used as template DNA to construct the deleted protein. Deletion of the α-5 helix of 14-3-3ε was produced by polymerase chain reaction (PCR) amplification of two fragments, both lacking the 60 bp sequence DNA of the α-5 helix. The primers used to make this deletion are listed in Table 1. Both fragments were digested with StyI restriction enzyme, followed by ligation. Wild-type and mutant PCR products were cloned separately into plasmid pEVP11 and the corresponding DNA sequences were verified by sequencing. The resulting plasmids were then used to transform yeast GG1259 cells by the lithium acetate procedure.

### Whole cell extract and western blotting

Cells were bead-beaten in lysis buffer (50 mM Tris, pH 7.5, 0.1% Triton X-100, 100 mM NaCl, 2.0 mM EDTA, 2.0 mM phenylmethylsulfonyl fluoride, 2.0 mM Sodium orthovanadate (Na3VO4), and 20 mM EGTA, 50 mM NaF), supplemented with 1 pill of protease inhibitors (Roche Molecular Biochemicals) before use. Lysate free of beads was microcentrifuged at maximum speed for 5 minutes at 4°C. The supernatant was mixed with equal volume of 2× protein sample buffer containing 100 mM dithiothreitol, and the samples were loaded onto a 12% SDS-polyacrylamide gel. Electrophoresis was carried out at 120 V for 90 minutes, and the gel contents were subsequently transferred onto Immobilon™ P transfer membrane. Immunoblotting was done by using anti-14-3-3ε antibody (T16) (Santa Cruz Biotechnology), anti-Bmh1 antibody (a gift from Dr van Heusden, Netherlands), and anti-Ras2 antibody (yC-19) (Santa Cruz Biotechnology).

### Electromobility-shift assay

#### Band-shift assays

Cruciform-containing DNA was constructed and end-labelled as described previously [7]. 20 μg of wild-type or mutant whole cell extracts (WCE) were incubated with ~3 ng of labelled cruciform DNA for 30 minutes on ice in binding buffer, as previously described [7]. The mixtures were subjected to 4% polyacrylamide gel electrophoresis (PAGE) at 180 V for 2 h. The gel was dried and exposed for autoradiography. R18 experiment was done as described previously [38] prior to the mobility shift assay. 20 μg of wild type 14-3-3ε yeast extract was incubated without or with increasing amounts (10, 50 and 100 μM) of R18 (Biomol International, LP) for 30 minutes at 30°C. Cruciform binding assay reagents were added and incubated on ice for 30 minutes before loading on the gel as described above.

#### Band-shift elution and immunoblotting

Wild-type and α5-deleted 14-3-3ε proteins were purified as previously described [8] with some modifications. In brief, 300 ng of labelled cruciform DNA [7] was used for binding either wild-type or mutant 14-3-3ε from 100 μg of their respective WCEs. Cruciform-protein complexes were subjected to PAGE in a 4% polyacrylamide gel at 180 V for 1.5 h. Each complex was eluted from the gel by isotachophoresis, as previously described [8]. Eluates were concentrated to 3 μg/μl with Microcon YM-10 concentrator (Millipore Corp.). Then, 30 μg of each band shift-eluted preparation were mixed with 1 × SDS sample buffer containing 100 mM dithiothreitol, and the samples were subjected to PAGE in a 12% SDS-polyacrylamide gel. The gel contents were transferred into Immobilon™ P transfer membrane, which was probed with anti-14-3-3ε antibody (T16) (Santa Cruz Biotechnology).

### Chromatin immunoprecipitation (ChIP) assay

#### *in vivo* cross-linking

*In vivo *cross-linking was carried out essentially as previously described [58]. In brief, 50 ml of wild-type and mutant cells were grown in YPD to *A*_595 _= 0.8–1.0. Cells were crosslinked with 1% formaldehyde and incubated for 1 hour at room temperature with gentle shaking. Cells were harvested by centrifugation at 1000 × *g *at 4°C for 15 minutes, the pellets were washed twice with ice-cold PBS, and then once with buffer I (50 mM Hepes/KOH (pH 7.5), 140 mM NaCl, 1 mM EDTA (pH 7.5), 1% (v/v) Triton X-100, and 0.1% (w/v) sodium deoxycholate).

#### Preparation of whole cell extracts

Pellets from cross-linked cells were resuspended in 250 μl of ice-cold buffer I containing protease inhibitors (1 mM phenylmethylsulfonyl fluoride and one tablet of protease inhibitors (Roche Molecular Biochemicals). The cell suspensions were lysed by bead-beating for 30 seconds and then chilled on ice for another 30 seconds for eight cycles. Beads were discarded and the lysates were sonicated for 15 seconds eight times, with 30 seconds intervals on ice between each pulse to chill the samples. After centrifugation at 14,000 rpm for 15 minutes at 4°C, protein concentrations of all samples were normalized with ice-cold buffer I. An aliquot of this supernatant served as the whole cell extract (WCE).

#### Immunoprecipitation and DNA isolation

2 mg total proteins of the WCE was precleared with 50 μl of protein G-agarose (Roche Molecular Biochemicals) for 1 h at 4°C and then incubated at 4°C for 12 h with 10 μl of either preimmune serum or 5 μg of anti-14-3-3ε antibody (Santa Cruz biotechnology). 50 μl of protein G-agarose was added, and the incubation was continued for 2 h. The precipitates were successively washed twice for 5 minutes at 4°C with 1 ml of each of the following buffers: ice-cold buffer I, ice-cold buffer II (50 mM Hepes/KOH (pH 7.5), 500 mM NaCl, 1 mM EDTA (pH 7.5), 1% (v/v) Triton X-100, and 1% (w/v) sodium deoxycholate); ice-cold buffer III (10 mM Tris-Cl (pH 8.0), 250 mM LiCl, 1 mM EDTA (pH 7.5), 0.5% (v/v) Nonidet P-40, and 0.5% (w/v) sodium deoxycholate); and ice-cold Tris/EDTA buffer (pH 7.6). Finally, the pellets were resuspended in 200 μl of extraction buffer (1% SDS/Tris/EDTA buffer). Samples were then incubated at 65°C overnight to reverse the protein-DNA cross-links, followed by 2 h incubation at 37°C with 50 μg of proteinase K (Roche Molecular Biochemicals). At the end, samples were processed to purify the DNA by passing them through QIAquick PCR purification columns (QIAGEN Inc., Valencia, CA).

#### PCR amplification of the co-immunoprecipitated DNA

The immunoprecipitated materials, WCE and genomic DNA, were used as templates in conventional PCR with Ready-To-Go PCR beads (Amersham Biosciences). Primers ARS307 (1 μM each; GENSETCorp.) (Tab. 1) were used to amplify a 370-bp DNA fragment from the yeast autonomous replication sequence ARS307 (GenBank™/EBI accession number X04219). An initial denaturation for 5 minutes at 94°C was followed by 35 cycles of denaturing for 30 seconds at 94°C, annealing for 30 seconds at 50°C, polymerization for 1 minute at 72°C, and a final extension for 10 minutes at 72°C. PCR products were separated on 1.5% agarose gel, visualized with ethidium bromide, and photographed with an Eagle Eye apparatus (Speed Light/BT Sciencetech-LT1000).

#### Real-time PCR amplification of the co-immunoprecipitated DNA

PCR reactions were carried out in 20 μl with one-two hundredth of the immunoprecipitated material, using LightCycler capillaries (Roche Molecular Biochemicals). Specific primers for the Real-time PCR (listed in Table 1) were added at 1 μM concentration. Genomic DNA was used to generate the standard curve. For the Real-time PCR reactions, an initial denaturation for 5 minutes at 95°C was followed by 35 cycles with denaturation for 15 seconds at 95°C; the annealing temperatures were used according to different fragments amplified (ARS307, Neg307, ARS1 or R2.5) for 10 seconds, followed by polymerization for 10 seconds at 72°C. The specificity of the amplified PCR products was assessed by performing a melting curve analysis after the PCR amplification.

### Plasmid stability assay

The assay was performed as described [59]. pARS-1 and pARS-2 were used separately to transform both wild type and mutant 14-3-3ε yeast strains, using the standard lithium acetate method. Cells were then grown to early log-phase in selective medium, SCM-His. The cultures were diluted to 2 10^5 ^cells/ml in YPD and grown for 10 generations. Equal amounts of cells were then plated on YPD and SCM-His plates, and plasmid loss rates were determined by counting colonies before and after incubation in YPD media. The stability value for each plasmid is an average of three independent experiments, each using colonies from a separate transformation.

## List of abbreviations

ACS, ARS consensus sequence; ARS, autonomous replication sequence ; CBP, cruciform-specific binding protein; ChIP, chromatin immunoprecipitation; IR, inverted repeat; ORC, origin recognition complex; pre-RC, prereplication complex; ts, temperature sensitive mutant; WCE, whole cell extract.

## Authors' contributions

**WY **carried out all the experiments described in the study except for the Uracil disruption cassette in GG1259 cells and the deletion of α5-helix in 14-3-3ε protein, and also conceived and designed the study and wrote the manuscript. **MC **carried out the GG1259 uracil cassette disruption and the α5-helix deletion in 14-3-3ε protein, and helped to conceive the study. **GBP **initially supervised and helped to conceive the study. **MZH **supervised the study and the manuscript. All authors, except GBP, have read and approved the final manuscript.

## Figures and Tables

**Table 1:** Sequences of primers used for the constructs and the LightCycler

**Primers**	**Sequences**
**α5-helix deletion**	
Fragment 1-F	5' CGGAATTCATGGATGATCCGAGAGGATCTG 3'
Fragment 2-F	5' GCTCTAGATCACTGATTTTCGTCTTCCAC 3'
Fragment 1-R	5' CGCCTTGGACTCGCCAGTGTTAGCTGC 3'
Fragment 2-R	5' GCCCAAGGCCACAGGAAACGACAGGAAGG 3'
**PCR of ARS307**	
ARS307-F	5' ATATTGCAATTACTTCTTCTCATGCAC 3'
ARS307-R	5' ATATTGCAATTACTTCTTCTCATGCAC 3'
PARS307-F	5' GGTAGGGATAATAATCTGTAATGAGGA 3'
PARS307-R	5' TATGTCGACGAGTGAGTTGGA 3'
**Real-Time PCR**	
RT-ARS307-F	5' GCAGTAGCACATGGACACATTCAC 3'
RT-ARS307-R	5' AGGGTTTCTGGAGAGGTAGGGATA 3'
NegARS307-F	5' ATTTTGTGGTGGATTGGAG 3'
NegARS307-R	5' GCTTGTTTGTGCAACTAAACG 3'
ARS1-F	5' GAAAATAGGTTATTACTGAGTAG 3'
ARS1-R	5' CCTGCGATGTATATTTTCCTG 3'
R2.5-F	5' CATCAATTGTGCACTCGGAC 3'
R2.5-R	5' GAACACGGCAATTGTAGGTGG 3'
